# Stability-Indicating Assay for the Determination of Pentobarbital Sodium in Liquid Formulations

**DOI:** 10.1155/2015/697937

**Published:** 2015-10-12

**Authors:** Myriam Ajemni, Issa-Bella Balde, Sofiane Kabiche, Sandra Carret, Jean-Eudes Fontan, Salvatore Cisternino, Joël Schlatter

**Affiliations:** Service Pharmacie, AP-HP Hôpital Jean-Verdier, Hôpitaux Universitaires de Paris-Seine-Saint-Denis, Avenue du 14 juillet, 93140 Bondy, France

## Abstract

A stability-indicating assay by reversed-phase high performance liquid chromatography (RP-HPLC) method was developed for the determination of pentobarbital sodium in oral formulations: a drug used for infant sedation in computed tomography (CT) or magnetic resonance imaging (MRI) scan. The chromatographic separation was achieved on a reversed-phase C18 column, using isocratic elution and a detector set at 214 nm. The optimized mobile phase consisted of a 0.01 M potassium buffer pH 3 and methanol (40 : 60, v/v). The flow rate was 1.0 mL/min and the run time of analysis was 5 min. The linearity of the method was demonstrated in the range of 5 to 250 *μ*g/mL pentobarbital sodium solution (*r* ^2^ = 0.999). The limit of detection and limit of quantification were 2.10 and 3.97 *μ*g/mL, respectively. The intraday and interday precisions were less than 2.1%. Accuracy of the method ranged from 99.2 to 101.3%. Stability studies indicate that the drug is stable to sunlight and in aqueous solution. Accelerated pentobarbital sodium breakdown by strong alkaline, acidic, or oxidative stress produced noninterfering peaks. This method allows accurate and reliable determination of pentobarbital sodium for drug stability assay in pharmaceutical studies.

## 1. Introduction

Pentobarbital sodium (5-ethyl-5-(1-methylbutyl)-2,4,6(1H,3H,5H)-pyrimidinetrione, sodium) is a psychoactive drug with short-acting sedative effects in adult and paediatric patients. However, it is not any longer marketed in Europe and in the United States. European drug agencies recently withdraw chloral hydrate, a widely used sedative drug, due to its adverse effects such as mutagenesis [1]. Pentobarbital sodium would be an alternative in paediatric sedative procedures such as in computed tomography or magnetic resonance imaging in infants. Clinical studies reported the effectiveness of pentobarbital sodium sedation and a decreased rate of adverse events as compared to chloral hydrate preimaging procedure [2–4]. Both drugs may also produce similar side effects including decreased oxygen saturation, vomiting, and respiratory depression [2–5]. The initial oral dose of sodium pentobarbital in sedation procedure for infants is usually 4-5 mg/kg with a maximum of 8 mg/kg. If the sedative response is not achieved, one additional 2 mg/kg oral dose can be administered [6]. A literature survey showed that only one liquid chromatography (HPLC) method is reported for the quantitative determination of pentobarbital sodium and some impurities in bulk drug substance and dosage forms with a chromatographic run of 30 min [7]. Drug crystallization could occur in 24 h when the pentobarbital sodium 50 mg/mL in 0.9% sodium chloride solution was further diluted to 10 mg/mL in repackaging polypropylene syringe [8]. More recently, Priest and Geisbuhler reported that injectable pentobarbital sodium was not degraded when stored in dark at room temperature using the HPLC method previously cited [9]. Here, we report a precise, accurate, and robust HPLC stability-indicating assay to assess pentobarbital sodium in oral/liquid compounding formulations which was validated for the first time with oxidative, alkali, and acidic breakdown and a chromatographic run time of 5 min. This assay was validated according to the International Conference on Harmonization [10].

## 2. Material and Method

### 2.1. Chemical and Reagents

Pharmaceutical pentobarbital sodium powder was supplied by Inresa (Bartenheim, France, lot 10026/1111B479). Phenobarbital sodium was used as an internal standard (IS) and was obtained from Sanofi Whintrop (Maisons-Alfort, France, lot 284). The compounding formulations Inorpha, Ora-Plus, Ora-Sweet, Ora-Sweet SF, Ora-Blend, and Ora-Blend SF were purchased from Inresa (Bartenheim, France, lots 4388549, 4469317, 4378457, 4287617, 4509679, and 4388553, resp.). The analytical grade methanol was obtained from Sigma-Aldrich (Chromasolv, St. Quentin Fallavier, France). Potassium dihydrogen phosphate was obtained from VWR Chemicals (Fontenay sous bois, France). Deionised water was purchased from Fresenius (Versylene, Sèvres, France).

### 2.2. HPLC Instrumentation and Conditions

The HPLC Dionex Ultimate 3000 system (Thermo Scientific, Villebon sur Yvette, France) contained an integrated solvent and degasser SRD-3200, an analytical pump HPG-3200SD, a thermostated autosampler WPS-3000TSL, a thermostated column compartment TCC-3000SD, and a diode array detector MWD-3000. Data acquisition (e.g., peak time and area) was carried out using in line Chromeleon software (v6.80 SP2) (Thermo Scientific). The eluent was monitored at 214 nm. Chromatographic separation was achieved at 25°C using a reverse phase Nova-Pak C18 column (60 Å, 4 *μ*m, 4.6 mm × 150 mm, Waters, Guyancourt, France). The mobile phase (0.01 M phosphate buffer pH 3: methanol; 40 : 60 v/v) was pumped at a flow rate of 1.0 mL/min. The injection volume was set at 25 *μ*L.

### 2.3. Preparation of Stock and Standards Solutions

#### 2.3.1. Pentobarbital Sodium Stock and Working Solutions

Pentobarbital sodium stock solution (1 mg/mL) was prepared by accurately weighing 100 mg. Volume was made up to the mark with deionised water in 100 mL volumetric flask. A working solution (0.1 mg/mL) was prepared by dilution of the stock solution. The solutions were stored at 2–8°C for 5 days.

#### 2.3.2. Preparation of the Internal Standard Solution

Phenobarbital sodium stock solution (1 mg/mL) was prepared by accurately weighing 100 mg. Volume was made up to the mark with deionised water in 100 mL volumetric flask. The stock solution was stored at 2–8°C for 5 days.

#### 2.3.3. Calibration Standards

Calibration standards at 5, 10, 20, 50, 100, and 200 *μ*g/mL were freshly prepared using either stock or working solution. These solutions contained IS at 20 *μ*g/mL.

#### 2.3.4. Quality Control Samples

Quality control solutions at 8, 15, 30, 80, and 150 *μ*g/mL containing IS (20 *μ*g/mL) were prepared extemporaneously.

### 2.4. Analytical Method Validation

#### 2.4.1. Linearity

Appropriate volumes of pentobarbital sodium stock (1 mg/mL) and working (100 *μ*g/mL) standard solutions were diluted with deionised water to yield 5, 10, 20, 50, 100, and 200 *μ*g/mL. Six replicates of each concentration were independently prepared and injected into the chromatograph. The linearity was determined by calculating a regression line from the plot of the peak area ratios of the drug and IS versus concentrations of the drug. Regression analyses were computed for pentobarbital sodium with Chromeleon software. The method was evaluated by determination of the correlation coefficient and intercept values according to the ICH guidelines.

#### 2.4.2. Limit of Detection and Limit of Quantification

Limit of detection (LOD) and limit of quantification (LOQ) of pentobarbital sodium assay were determined by calibration curve method. Solutions of pentobarbital sodium were prepared in linearity range and injected in triplicate. Average peak area of three analyses was plotted against concentration. LOD and LOQ were calculated by using the following equations: LOD = (3.3 × *S*_*yx*_)/*b*, LOQ = (10.0 × *S*_*yx*_)/*b*, where *S*_*yx*_ is residual variance due to regression; *b* is the slope.

#### 2.4.3. Precision

The intraday precision was determined by measuring quality control samples of 8, 15, 30, 80, and 150 *μ*g/mL of pentobarbital sodium, injected six times on the same day. The intermediate precision was estimated by injecting quality control samples prepared at the same concentrations on three different days by different operators. The peak area ratios of all injections were taken and standard deviation, % relative standard deviation (RSD), was calculated.

#### 2.4.4. Accuracy

Accuracy is tested by the standard addition method at different levels: 25, 50, 80, 100, and 120%. The mean recovery of pentobarbital sodium of the target concentration (50 *μ*g/mL) was calculated and accepted with 100 ± 2%.

#### 2.4.5. Robustness

HPLC conditions were slightly modified to evaluate the analytical method robustness. These changes (see Table 1) included the flow rate, the detection wavelength, the column temperature, or the methanol proportion in the mobile phase.

#### 2.4.6. Forced Degradation Study

Alkaline, acidic, and oxidative stress and direct exposure to sunlight were carried out as reported in Table 2. No internal standard was added in the forced degradation study.

*(1) Alkali Hydrolysis*. Ten mL of pentobarbital stock solution was mixed in a flask with 1 N sodium hydroxide (4 mL) for 1 h at 50°C. Before analysis, the solution was cooled at room temperature and neutralized with hydrochloric acid. The solution was completed with deionised water to reach a targeted concentration of 50 *μ*g/mL in a volumetric flask.

*(2) Acid Hydrolysis*. Ten mL of pentobarbital stock solution was mixed in a flask with 1 N hydrochloride acid (4 mL) for 1 h at 50°C. Before analysis, the solution was cooled at room temperature and neutralized with sodium hydroxide. The solution was completed with deionised water to reach a targeted concentration of 50 *μ*g/mL in a volumetric flask. 

*(3) Oxidative Stress*. Ten mL of the pentobarbital stock solution was mixed with 1 mL of 3% hydrogen peroxide and stored at 50°C for 1 h. The solution was cooled and completed with deionised water until the volumetric flask mark to reach a targeted concentration of 50 *μ*g/mL. 

*(4) Sunlight Degradation*. Ten mL of the pentobarbital stock solution was transferred into a 200 mL volumetric flask and exposed to direct sunlight for 5 days at room temperature. The solution was completed to the flask mark with deionised water. 

*(5) Thermal Degradation*. Ten mL of stock solution was transferred into volumetric flask (200 mL) and kept in air dry oven at 105°C for 5 h. Then, the solution was cooled and completed to the flask mark with deionised water. 

*(6) Hydrolytic Degradation*. Ten mL of pentobarbital stock solution was transferred into a volumetric flask and mixed with 10 mL of deionised water. The solution was heated on water bath for 1 h. Then, the solution was cooled and completed until the 200 mL flask mark with water to reach a hypothetical target concentration of 50 *μ*g/mL.

## 3. Results and Discussion

### 3.1. Analytical Development Method

**Figure 1:**
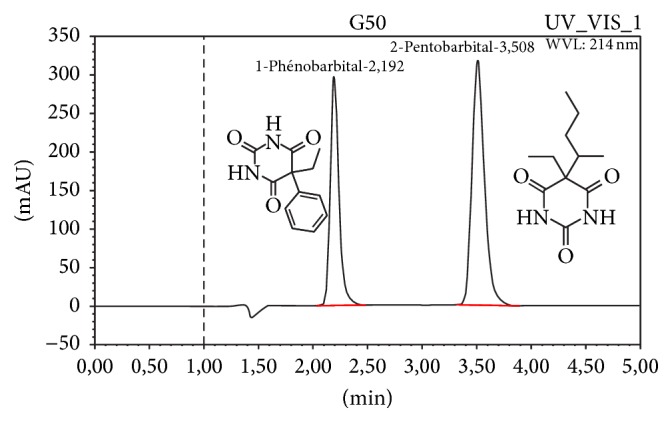
Typical chromatogram of pentobarbital sodium and internal standard and their chemical structures.

In order to achieve optimum separation, pentobarbital sodium and IS were injected into different mobile phase solutions mixing phosphate buffer and acetonitrile or phosphate buffer and methanol at different proportions, 70 : 30, 60 : 40, 50 : 50, and 40 : 60, and pH values, 7, 5, or 3. The retention time and tailing factor along with resolution factor were recorded. As the pKa of pentobarbital is reported to be 8.1, mobile phase with pH 3 was selected. Using the Nova-Pak C18 column, pentobarbital sodium and IS were eluted at 3.5 and 2.2 min, respectively. Column temperature (22–26°C) was found to be not a critical factor of this analysis. The optimum UV absorption of the drug was obtained at 214 nm as there was no interference from excipients present in oral compounding formulations. A typical chromatogram obtained with the present method is depicted in Figure 1.

### 3.2. Method Validation

#### 3.2.1. Linearity

The linearity range of pentobarbital sodium was in the interval of 5–200 *μ*g/mL. These were represented by a mean linear regression equation as follows: *y* = 0.0291*x* + 0.0378 with 0.9998 correlation coefficient and regression line was established by least squares method (Table 3).

#### 3.2.2. Limit of Detection (LOD) and Limit of Quantification (LOQ)

The determined values of LOD and LOQ were 2.103 and 3.979 *μ*g/mL calculated using slope and Y-intercept as per ICH guideline.

#### 3.2.3. Precision

The results were obtained for the intraday and interday precision of the method, expressed as RSD values. As shown in the table, the intraday and interday RSD were <2.1% for all concentrations tested in different situations studied (Table 4).

#### 3.2.4. Accuracy

The percentage recoveries were found to be 99.2 to 101.3% (Table 5). The results of the recovery studies undoubtedly demonstrate accuracy of the proposed method.

#### 3.2.5. Specificity

The specificity was estimated by spiking compounding vehicles as Ora-Plus, Ora-Sweet, Ora-Sweet SF, Ora-Blend, Ora-Blend SF, and Inorpha into a preweighed quantity of drug. The specificity study was carried out to check the interference from the excipients used in these vehicles. The chromatogram showed peak for pentobarbital sodium without any interfering peak.

#### 3.2.6. Robustness

The robustness of the method was illustrated by getting the resolution (*R*_*s*_), the tailing factor of the drug (*T*_*f*_-D), the tailing factor of the internal standard (*T*_*f*_-IS), and the number of plates when flow rate, wavelength detection, column temperature, and methanol proportion were slightly changed (Table 1). Table 1 shows that the percent recoveries of pentobarbital sodium were good under most conditions except for the wavelength condition at 225 nm. The deliberate changes in the method do not affect the resolution, tailing factors of drug and IS, and number of plates significantly (Table 1).

#### 3.2.7. System Suitability Parameters

The system suitability tests were studied before performing the validation and the calculated parameters are within the acceptance criteria. The capacity factor was 1.39, the resolution was 7.65, the selectivity was 1.6, the number of theoretical plates was 5550, the tailing factor (*T*_*f*_-D) of drug was 1.30, the tailing factor of internal standard (*T*_*f*_-IS) was 1.35, and the RSD of repeatability of injection were <0.3%. Hence, the proposed method was successfully applied to routine analysis.

#### 3.2.8. Stability of Sample

Stability of the sample solution was established by storage of the sample solution at refrigerator (2–8°C) for 21 days and at room temperature for 24 h. The results from the solution stability experiments confirmed that the sample solution was stable for up to 21 days at refrigerator and during assay determination.

#### 3.2.9. Forced Degradation Study

**Figure 2:**
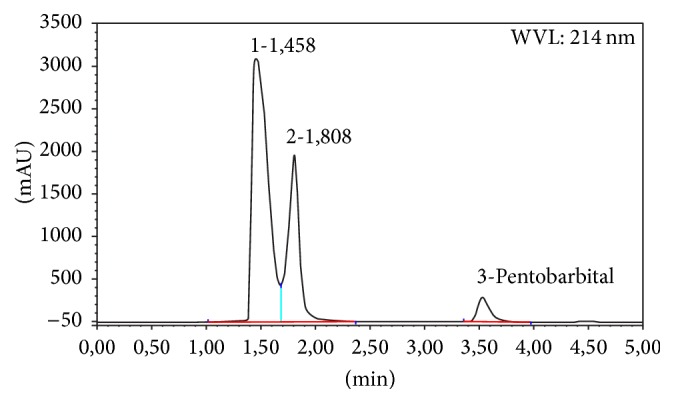
Chromatogram of 12 N NaOH treated pentobarbital sodium at 50°C for 48 h.

**Figure 3:**
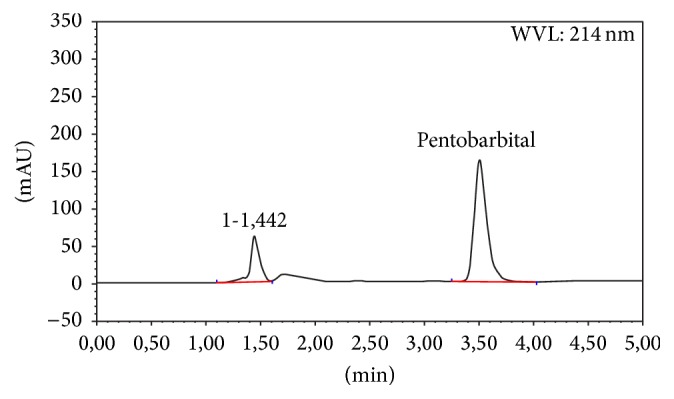
Chromatogram of 3% hydrogen peroxide treated pentobarbital sodium at 50°C for 48 h.

Forced degradation studies were performed to demonstrate the stability-indicating capability of the proposed HPLC method (Table 2). No degradation of pentobarbital sodium exposed to 1 N HCl, 1 N NaOH, and direct sunlight was observed. Due to this particular stability, high acidic and alkaline stress conditions were performed using 10 N NaOH and 12 N HCl at 50°C for 48 h. A chromatogram of high alkaline hydrolysis performed at 50°C for 48 h showed degradation product peaks at retention times 1.46 and 1.81 min (Figure 2). A chromatogram of oxidative stress performed at 50°C for 48 h showed degradation product peak at retention time 1.44 min (Figure 3). The compound was stable at high temperature (50°C) and in aqueous solution. These statements are in agreement with the 6.5% loss of potency described by Gupta [8] in its pentobarbital preparation boiled for 1.5 h and the complete degradation of pentobarbital in 30 days using combination of high pH and 20% formaldehyde described by Gannet et al. [11].

## 4. Conclusion

This rapid and simple RP-HPLC method was successfully developed for the determination of pentobarbital sodium stability in water solution. The developed analytical method is precise, accurate, and linear. Forced degradation data proved that the method is specific for the analyte and free from the interference of blank and unknown degradation products. The method is suitable for the analysis of stability samples and the routine analysis of pentobarbital sodium in formulations.

## Figures and Tables

**Table 1:** Robustness.

Parameters	Modification	% recovery	*R*_*s*_	*T*_*f*_-D	*T*_*f*_-IS	Plates
Flow rate (mL/min)	1.1	100.3	8.06	1.30	1.34	5503
	1.2	100.2	7.73	1.26	1.27	5155
	1.3	100.2	7.46	1.19	1.38	4779

Wavelength of detection (nm)	218	105.0	8.46	1.27	1.42	6042
	220	103.4	8.43	1.33	1.35	5969
	225	78.5	8.41	1.31	1.42	5848

Column temperature (°C)	25	100.1	8.22	1.30	1.46	5828
	27	100.1	8.02	1.35	1.34	5856
	30	100.0	7.80	1.36	1.32	5944

Methanol in mobile phase	−0.2%	100.0	8.42	1.33	1.38	6002
	+0.2%	100.0	7.33	1.39	1.42	5549

*R*_*s*_: resolution; *T*_*f*_-D: tailing factor of the drug; *T*_*f*_-IS: tailing factor of the internal standard.

**Table 2:** Forced degradations studies.

Stress conditions	% remaining	% degradation	Retention time of degraded products
Acidic stress (1 N HCl, 50°C, 1 h)	102.2	—	0.0
High acidic stress (12 N HCl, 50°C, 48 h)	31.8	68.2	0.0
Alkaline stress (1 N NaOH, 50°C, 1 h)	98.4	1.6	0.0
High alkaline stress (10 N NaOH, 50°C, 48 h)	89.8	10.2	1.46, 1.81
Oxidative stress (3%, 50°C, 48 h)	50.9	49.1	1.44
Thermal stress (50°C, 5 days)	89.4	10.6	0.0
High thermal stress (100°C, 1 h)	19.9	80.1	0.0
Direct sunlight (48 h)	95.1	4.9	0.0
Aqueous stability (after 21 days)	99.7	0.3	0.0

**Table 3:** Linearity data of the developed method.

Initial conc. (*μ*g/mL)	Mean peak area ± S.D. (pentobarbital) (*n* = 6)	Mean peak area (IS)	Mean peak ratio	Actual conc. (*μ*g/mL)	% assay
5	4.285 ± 0.020	27.805 ± 0.192	0.154 ± 0.001	3.99 ± 1.29	79.8
10	8.637 ± 0.050	27.897 ± 0.284	0.310 ± 0.001	9.33 ± 1.29	93.3
20	17.098 ± 0.143	27.186 ± 1.093	0.630 ± 0.031	20.34 ± 1.19	101.7
50	42.565 ± 1.147	27.983 ± 0.376	1.521 ± 0.028	50.96 ± 1.20	101.9
100	83.084 ± 1.149	27.856 ± 0.111	2.983 ± 0.047	101.19 ± 1.14	101.2
200	164.194 ± 0.401	28.135 ± 0.253	5.836 ± 0.048	199.26 ± 1.13	99.6
*y* = 0.0291*x* + 0.0378, *r* ^2^ = 0.9998					

**Table 4:** Precision study of the method.

Nominal conc. (*μ*g/mL)	Intraday precision			Interday precision		
	Calculated conc. (*μ*g/mL), mean ± SD	Accuracy (%bias)	RSD	Calculated conc. (*μ*g/mL), mean ± SD	Accuracy (%bias)	RSD
8	8.273 ± 0.009	3.42	0.11	8.181 ± 0.103	2.27	1.25
15	15.280 ± 0.143	1.86	0.94	15.052 ± 0.208	0.35	1.38
30	30.748 ± 0.632	2.49	2.06	30.355 ± 0.386	1.18	1.27
80	81.738 ± 0.602	2.17	0.74	81.257 ± 1,218	1.57	1.50
150	153.569 ± 0.328	2.38	0.21	152,278 ± 2,139	1.52	1.41

**Table 5:** Accuracy of the method.

Standard (*μ*g/mL)	Added		Found (*μ*g/mL)	% recovery	RSD
	%	*μ*g/mL	Mean ± SD, *n* = 6	Mean ± SD	
50	25	62.5	63.29 ± 0.48	101.27 ± 0.77	0.77
50	50	75	74.78 ± 0.56	99.71 ± 0.75	0.75
50	80	90	89.55 ± 2.61	99.49 ± 2.90	2.90
50	100	100	99.23 ± 1.22	99.23 ± 1.22	1.22
50	120	110	110.59 ± 0.28	100.53 ± 0.25	0.25
